# Evaluation of reproductive barriers contributes to the development of novel interspecific hybrids in the *Kalanchoë* genus

**DOI:** 10.1186/s12870-014-0394-0

**Published:** 2015-01-21

**Authors:** Katarzyna Kuligowska, Henrik Lütken, Brian Christensen, Ib Skovgaard, Marcus Linde, Traud Winkelmann, Renate Müller

**Affiliations:** Department of Plant and Environmental Sciences, Faculty of Science, University of Copenhagen, Højbakkegård Allé 9-13, DK-2630 Taastrup, Denmark; AgroTech A/S, Inst. f. Agri Technology and Food Innovation, Højbakkegård Allé 21, DK-2630 Taastrup, Denmark; Department of Mathematical Sciences, Faculty of Science, University of Copenhagen, Universitetsparken 5, DK-2100 København Ø, Denmark; Institute for Plant Genetics, Leibniz Universität Hannover, Herrenhaeuser Str. 2, D-30419 Hannover, Germany; Institute of Horticultural Production Systems, Leibniz Universität Hannover, Herrenhaeuser Str. 2, D-30419 Hannover, Germany

**Keywords:** AFLP markers, Chromosome number, Cross-compatibility, Endosperm development, Genetic distance, Pollen tube growth, Pre-zygotic barrier, Post-zygotic barrier

## Abstract

**Background:**

Interspecific hybridization is a useful tool in ornamental breeding to increase genetic variability and introduce new valuable traits into existing cultivars. The successful formation of interspecific hybrids is frequently limited by the presence of pre- and post-fertilization barriers. In the present study, we investigated the nature of hybridization barriers occurring in crosses between *Kalanchoë* species and evaluated possibilities of obtaining interspecific hybrids.

**Results:**

The qualitative and quantitative analyses of pollen tube growth *in situ* were performed following intra- and interspecific pollinations. They revealed occurrence of pre-fertilization barriers associated with inhibition of pollen germination on the stigma and abnormal growth of pollen tubes. Unilateral incongruity related to differences in pistil length was also observed. The pollen quality was identified as a strong factor influencing the number of pollen tubes germinating in the stigma. In relation to post-fertilization barriers, endosperm degeneration was a probable barrier hampering production of interspecific hybrids. Moreover, our results demonstrate the relation of genetic distance estimated by AFLP marker analysis of hybridization partners with cross-compatibility of *Kalanchoë* species. At the same time, differences in ploidy did not influence the success of interspecific crosses.

**Conclusions:**

Our study presents the first comprehensive analysis of hybridization barriers occurring within *Kalanchoë* genus. Reproductive barriers were detected on both, pre- and post-fertilization levels. This new knowledge will contribute to further understanding of reproductive isolation of *Kalanchoë* species and facilitate breeding of new cultivars. For the first time, interspecific hybrids between *K. nyikae* as maternal plant and *K. blossfeldiana* as well as *K. blossfeldiana* and *K. marnieriana* were generated.

**Electronic supplementary material:**

The online version of this article (doi:10.1186/s12870-014-0394-0) contains supplementary material, which is available to authorized users.

## Background

Interspecific hybridization, as a mean to increase genetic variability and introduce new valuable traits, has been carried out in many cultivated plants. In the field of ornamental breeding, interspecific hybridization is considered to be one of the most useful strategies to develop new cultivars. This technique has been successfully used as a breeding tool in ornamental plants including *Rosa* [1]*,* C*hrysanthemum* [2]*, Dianthus* [3], *Lilium* [4] and *Rhododendron* [5].

Crosses among plants belonging to different species are naturally occurring phenomena when distributions of species overlap [6]. There are, however, processes that ensure reproductive isolation of distinct species. Hybridization barriers that hamper the development of interspecific hybrids are typically recognized as pre-fertilization barriers and post-fertilization barriers, depending on the time point of their action during the hybridization process. Pre-fertilization barriers prevent mating and fertilization. They include lack of stigma receptivity or pollen viability at the time of pollination or abnormal growth of pollen tubes in the style and ovary of recipient partner [7]. Successful formation of hybrid plants may also be limited by post-fertilization barriers. They include embryo and endosperm abortion, abnormal growth and unviability of hybrids or their sterility [7].

The *Kalanchoë* genus belongs to the Crassulaceae family and consists of around 140 species, mostly succulents. *Kalanchoë* species are mainly native to Madagascar and the east coast of Africa. The genus is divided into two sections Kalanchoë and Bryophyllum, based primarily on flower characteristics and an ability to viviparous plant formation [8]. The first plant of *Kalanchoë blossfeldiana* was introduced to Europe from Madagascar in 1924. Development of new cultivars was initiated in 1930’s, however, it resulted from selection within the progeny of a single plant. Interspecific hybridization of *K. blossfeldiana* was successfully conducted in 1939, when the first interspecific hybrid with *K. flammea* was obtained. From the beginning, breeding of *Kalanchoë* cultivars was focused on compactness and flower characteristics such as color and double flowers [9]. Thus, little variation is present in respect to other plant features.

*K. blossfeldiana* and its interspecific hybrids are popular ornamental plants. They are used both as flowering potted plants and garden plants. *Kalanchoë* cultivars are economically important with an annual production of 41 million plants in Denmark in 2012 [10]. In the Netherlands, 77 million plants were produced with turnover of 55 million euros in 2012 [11].

Within the *Kalanchoë* genus, there are other species with various interesting features like different plant architecture, flower shape or leaf morphology. Thus, wild species within the *Kalanchoë* genus represent essential genetic resources that may increase the restricted genetic basis of modern *K. blossfeldiana* cultivars.

There have been previous reports on interspecific hybridization in the *Kalanchoë* genus*.* Intrasectional hybridization resulted in hybrid progeny among *K. blossfeldiana* and several other *Kalanchoë* species such as *K. citrina, K. farinacea, K. garambiensis, K. nyikae, K. pumila, K. spathulata* [12]. Successful intersectional hybridization was previously reported among *K. blossfeldiana* and *K. daigremontiana, K. laxiflora, K. pubescens,* as well as *K. spathulata* and *K. laxiflora* [13]. However, a systematic investigation of general cross-compatibility among *Kalanchoë* species is missing and the nature of hybridization barriers occurring during hybridization is unknown. Information about chromosome numbers in the genus is also limited.

The objectives of this study were to identify the nature of hybridization barriers occurring in reciprocal crosses among selected *Kalanchoë* species. The influence of plant genotype, chromosome numbers determined by DAPI staining and genetic similarity assessed by AFLP markers on cross-compatibility and production of novel interspecific hybrids within the *Kalanchoë* genus was evaluated. The analysis of interspecific crosses revealed the hybridization barriers during both, the pre- and post-fertilization phase of reproductive process. They were related to parental divergence and influenced by the specific genetic background of parental plants. The obtained interspecific progeny exhibited intermediate phenotypes typical for hybrids. In addition, transgressive segregation was also observed in some of the hybrid lines.

## Methods

### Plant material

**Figure 1:**
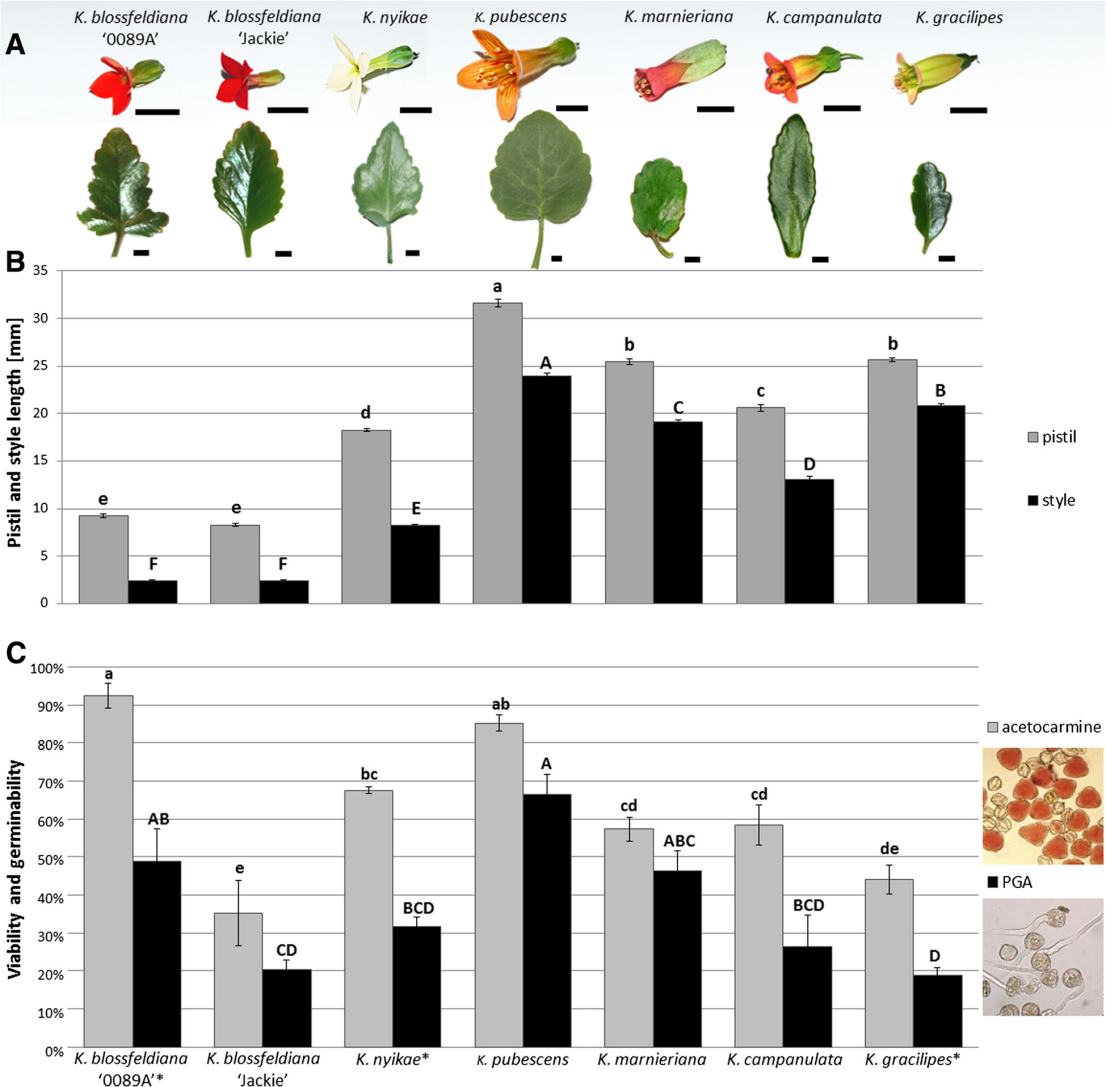
**Characterization of** ***Kalanchoë*** **genotypes. (A)** Flower and leaf morphologies of different *Kalanchoë* species and cultivars. *Scale bars*: 1.0 cm. **(B)** Pistil and style length. Values present means (±S.E.), n = 20. Values followed by different letters (small letters for pistils and capital letters for style) are significantly different (*P* ≤0.05) according to Tukey’s honestly significant difference test. **(C)** The viability and germinability of pollen samples. Pollen viability was determined by staining with 1% acetocarmine, pollen germinability was determined by incubation in liquid germination medium. Values presented are means (±S.E.), n = 3. Values followed by different letters (small letters for viability and capital letters for germinability) are significantly different (*P* ≤0.05) according to Tukey’s honestly significant difference test, genotype name followed by asterisks indicates values significantly different (*P* ≤0.05) between two performed pollen analyses according to t-test.

Seven genotypes belonging to six *Kalanchoë* species were used in the experiment; two cultivars of *K. blossfeldiana* VAN POELLN. ‘Jackie’ and ‘0089A’ and five wild species: *K. nyikae* BAK., *K. pubescens* BAK., *K. marnieriana* JACOBS., *K. campanulata* (BAK.) BAILL., *K. gracilipes* (BAK.) BAILL. The taxonomic position and origin of the species are shown in Table 1. Leaf and flower morphologies are presented in Figure 1A.

**Table 1:** **Overview of parental plants**

**Species**	**Section**	**Origin**
*K. blossfeldiana* VAN POELLN. cv. ‘0089A’	Kalanchoë	Madagascar
*K. blossfeldiana* hybrid cv. ‘Jackie’		
*K. nyikae* BAK.	Kalanchoë	Africa
*K. pubescens* BAK.	Bryophyllum	Madagascar
*K. marnieriana* JACOBS.	Bryophyllum	Madagascar
*K. campanulata* (BAK.) BAILL.	Bryophyllum	Madagascar
*K. gracilipes* (BAK.) BAILL.	Bryophyllum	Madagascar

Plants were established from cuttings obtained from the greenhouse nursery Knud Jepsen A/S, Hinnerup, Denmark. The plants were cultivated in the greenhouse from April 2011 until April 2013 under standard conditions (22/18 ± 4°C day/night) and irrigated every second day with standard fertilizer (Pioner NPK Makro 14-3-23). Plants were kept under long day conditions with a photoperiod of 16/8 h, day/night with additional light of 260 μmol s^−1^ m^−2^ (Philips Master SON-T PIA Green Power 400 W). For flower induction plants were transferred to short day conditions (8/16 h, day/night) for about 10 weeks.

### Determination of chromosome number

For cytological observations the shoot tips with three leaf pairs were excised from parental plants and rooted in hydroponics for approx. 2 weeks. The root tips of induced adventitious roots were collected and prepared according to Lütken et al. [14]. DAPI-stained chromosomes were examined under the fluorescence microscope (Motic BA410, Wetzlar, Germany; excitation filter BP 350 nm) equipped with a digital camera (Moticam Pro 252, Wetzlar, Germany). A minimum of five cells with clearly visible chromosomes was observed for each genotype from at least two different plants.

### DNA extraction and AFLP analysis

Approximately 2 g of fresh leaf or flower material was immersed in liquid nitrogen and ground into a fine powder. Total genomic DNA was extracted from plants following the CTAB method after Doyle and Doyle [15]. Approx. 10 ml of CTAB extraction buffer (100 mM Tris–HCl pH 8.0, 2% (w/v) CTAB, 20 mM Na_2_EDTA, 1.4 M NaCl, 3% (w/v) PVPP and 1% (v/v) β-mercaptoethanol) and RNase A (10U) were added. Samples were incubated for 1 h at 65°C with occasional shaking. Afterwards, samples were homogenized with 1 vol. of chloroform:isoamyl alcohol (24:1) for 20 min and centrifuged for 25 min at 4250 × g. The supernatant was transferred to a new tube and chloroform:isoamyl alcohol extraction was repeated. Subsequently, 1 vol. of ice-cold isopropanol was added to the supernatant and samples were left for DNA precipitation overnight at −20°C. On the next day, samples were centrifuged for 5 min at 4250 × g and the pellet was washed with 96% v/v and 70% v/v ethanol and dried. DNA was dissolved in ddH_2_O. DNA concentration and quality was estimated in comparison to known DNA concentrations of λ-DNA in a 1% agarose gel following electrophoresis.

The AFLP method was performed essentially as described by Vos et al. [16] with minor modifications. The genomic DNA (100 ng) was digested with the restriction enzymes *Hind*III (9 U) and *Mse*I (3.6 U) overnight at 37°C. The *Hind*III adapter (5’-AGCTGGTACGCAGTCTAC) (2.5 pmol) and *Mse*I adapter (5’-ACTCAGGACTCAT) (25 pmol) were ligated to the restriction fragments with 0.25 U of T4-DNA ligase at 37°C for 3.5 h. For the pre-amplification, primers homologues to the adapter and the restriction site sequences containing one selective nucleotide (*Hind*III) or no selective nucleotide (*Mse*I) were used. Selective amplification was carried out using *Hind*III/*Mse*I with three selective nucleotides at the 3’-end. The 5’-end of the *Hind*III primer was IRD700 labeled. To estimate the reproducibility of the AFLP marker pattern, two independent analyses starting from the restriction until the selective amplification were performed for the three genotypes *K. blossfeldiana* ‘0089A’, *K. pubescens* and *K. gracilipes*. No deviating banding patterns were observed between the two replicates using -AAC/-CCA (*Hind*III/ *Mse*I) primer combinations.

All AFLP reactions for the estimation of the genetic diversity and the hybrid identification were performed on samples from two independent DNA extractions per genotype. The PCR fragments were separated using 6% denaturing polyacrylamide gels under standard conditions. The gels were scanned with an automatic LI-COR DNA sequencer (LI-COR Global IR2 4200LI-1 Sequencing System, LI-COR) for fragment detection. Only reproducible bands for the two independent biological replicates of each genotype were included in the further analysis.

### Analysis of genetic diversity

The banding patterns were assessed by visual inspection and transformed into a 0/1 matrix for each DNA fragment. Genetic distances were calculated using the FAMD 1.3 software package (http://www.famd.me.uk/famd.html). The pairwise distances between the analyzed plants were calculated using Jaccard similarity index. A cluster analysis was performed using the Neighbour-Joining method (Saitou and Nei 1987) [17]. To evaluate the robustness of the dendrogram, a bootstrap analysis (Felsenstein 1985) [18] with 1000 replicates was conducted. The dendrogram was constructed using FAMD (Schlüter and Harris 2006) [19] and displayed using Mega 5.2.2 (http://www.megasoftware.net/).

### Sexual hybridizations

Intraspecific and interspecific crosses were performed on a total of 29 cross-combinations (7 self-pollinations, 2 intraspecific – crosses of the two *K. blossfeldiana* genotypes and 20 interspecific crosses, Table 2). Pollination was carried out from April 2012 to April 2013. Interspecific crosses included reciprocal pollination between each of the two cultivars of *K. blossfeldiana* and the five other species within the *Kalanchoë* genus.

**Table 2:** **Overview of intra- and interspecific crosses among** ***Kalanchoë*** **species**

**Genetic distance**		**♀**	**♂**	**No. of flowers pollinated**	**Mean no. of seeds per capsule (SE)** ^**a**^	**Germination percentage** ^**b**^	**Total no. of seedlings**	**Total no. of plants**	**Seedling survival [%]**	**Total no. of hybrids**	**Hybrids [%]**
Self-pollination		*K. b.* ^1^ *‘*0089A’	*K. b. ‘*0089A’	10	40.8 (8.4)	52	418	400	96	NA^e^	NA
		*K. b.* ‘Jackie’	*K. b.* ‘Jackie’	10	5.0 (1.6)	40	3	3	100	NA	NA
		*K. nyikae*	*K. nyikae*	10	>50.0	67	1520	249^d^	83	NA	NA
		*K. pub.* ^2^	*K. pub.*	10	>50.0	98	398	390	98	NA	NA
		*K. mar.* ^3^	*K. mar.*	10	>50.0	63	369	254	69	NA	NA
		*K. cam.* ^4^	*K. cam.*	10	12.2 (3.8)	98	71	71	100	NA	NA
		*K. gra.* ^5^	*K. gra.*	10	0.0	-^c^	0	-	-	-	-
Intra-specific	0.312	*K. b. ‘*0089A’	*K. b.* ‘Jackie’	10	28.0 (6.3)	93	328	320	98	ND^f^	ND
		*K. b.* ‘Jackie’	*K. b. ‘*0089A’	10	11.8 (3.2)	63	62	60	97	ND	ND
Intra-sectional	0.695	*K. b. ‘*0089A’	*K. nyikae*	84	28.2 (4.7)	87	1521	213^d^	71	213	100.0
		*K. nyikae*	*K. b. ‘*0089A’	36	4.3 (1.0)	88	167	131	78	11	9.2
	0.695	*K. b.* ‘Jackie’	*K. nyikae*	42	6.0 (1.7)	88	234	121	52	121	100.0
		*K. nyikae*	*K. b.* ‘Jackie’	60	0.2 (0.1)	-	0	-	-	-	-
Inter-sectional	0.73	*K. b. ‘*0089A’	*K. pub.*	95	2.9 (0.9)	65	149	73	49	73	100.0
		*K. pub.*	*K. b. ‘*0089A’	94	0.0	-	0	-	-	-	-
	0.77	*K. b. ‘*0089A’	*K. cam.*	136	0.0	-	0	-	-	-	-
		*K. cam.*	*K. b. ‘*0089A’	77	0.0	-	0	-	-	-	-
	0.781	*K. b. ‘*0089A’	*K. mar.*	76	2.5 (1.0)	37	94	89	95	23	25.8
		*K. mar.*	*K. b. ‘*0089A’	54	0.0	-	0	-	-	-	-
	0.785	*K. b. ‘*0089A’	*K. gra.*	122	0.0	-	0	-	-	-	-
		*K. gra.*	*K. b. ‘*0089A’	110	0.0	-	0	-	-	-	-
	0.733	*K. b.* ‘Jackie’	*K. pub.*	101	1.3 (0.4)	67	94	46	49	46	100.0
		*K. pub.*	*K. b.* ‘Jackie’	44	0.0	-	0	-	-	-	-
	0.764	*K. b.* ‘Jackie’	*K. cam.*	160	0.0	-	0	-	-	-	-
		*K. cam.*	*K. b.* ‘Jackie’	60	0.0	-	0	-	-	-	-
	0.783	*K. b.* ‘Jackie’	*K. gra.*	92	0.0	-	0	-	-	-	-
		*K. gra.*	*K. b.* ‘Jackie’	51	0.0	-	0	-	-	-	-
	0.801	*K. b.* ‘Jackie’	*K. mar.*	68	0.2 (0.2)	-	0	-	-	-	-
		*K. mar.*	*K. b.* ‘Jackie’	112	0.0	-	0	-	-	-	-

^a^n = 10; ^b^n = 60; ^c^not applicable; ^d^out of 300 transferred seedlings; ^e^not applicable; ^f^no data available.

^1^ *K. b. – K. blossfeldiana;* ^2^ *K. pub. – K. pubescens;* ^3^ *K. mar. – K. marnieriana;* ^4^ *K. cam. – K. campanulata;* ^5^ *K. gra. – K. gracilipes*.

The table presents seed production, germination and hybrid production of intraspecific crosses and interspecific crosses of two *K. blossfeldiana* cultivars that are ordered according to genetic distance.

Flowers of the *Kalanchoë* species were emasculated in the bud stage, 3 to 1 day prior to anthesis. Anthers (and a part of corolla in *K. blossfeldiana* and *K. nyikae*) were removed using forceps. Flowers were pollinated in the expanded sticky stage of stigma with fresh pollen using a brush. Pollinated flowers were used either for examination of pollen tube growth *in situ* or left for the seed to mature for later collection.

### Pollen quality

#### Pollen viability

Pollen was collected at the point of anther dehiscence i.e. the day of flower opening before noon. Pollen of 10 flowers was immersed in a drop of 1% (w/v) acetocarmine solution [20]. Pollen was examined under the light microscope (Leitz DMRD, Leica, Germany) and pollen grains were scored: stained red as viable and unstained as unviable.

#### *In vitro* pollen germination

The assessment of germinability *in vitro* was conducted in liquid medium after Taylor [21]: 0.04% (w/v) H_3_BO_3_, 0.04% (w/v) Ca(NO_3_)_2_, 0.07% (w/v) MnSO_4_ with 10% sucrose concentration. The selection of culture medium was made following a preliminary screening (data not shown). Pollen of 10 flowers were immersed in this medium on glass slides and covered with cover glass. After 2 h of incubation (dark, room temperature) pollen grains were analyzed by the light microscope (Leitz DMRD, Leica, Germany). Pollen grains were classified as able to germinate when the pollen tube length exceeded the diameter of the pollen grain.

At least 100 pollen grains were analyzed per species. Each experiment was performed once in three technical replicates.

### Pollen tube growth *in situ*

Pollen tube growth was examined for all interspecific and intraspecific crosses at two time points: 24 and 48 hours after pollination. For each time point, carpels of 10 flowers were harvested and fixed in a 3:1 solution of absolute ethanol and glacial acetic acid. After 24 h the pistils were transferred to 70% (v/v) ethanol and stored at 4°C until use. Pistils were softened in 1 M NaOH for 25–35 min at 55°C and stained overnight with 0.1% (w/v) aniline blue in 50 mM KPO_4_ buffer in the dark. Subsequently, pistils were placed in 40% (v/v) glycerol and squashed under cover glass. Microscopic slides were stored at 4° until examination. Pollen tubes were examined under the fluorescent microscope (Leitz DMRD, Leica, Germany; excitation filter BP 340–360 nm) equipped with a digital camera (Leica DFC420, Leica, Germany).

**Figure 2:**
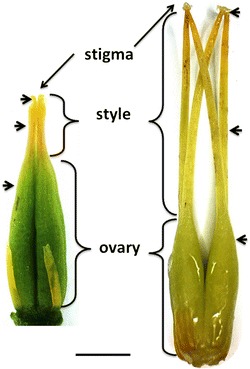
**The morphology of pistils.** Arrows indicate places in which pollen tubes were examined. *Scale bar:* 2.0 mm.

Since the size of the pistil differed significantly among the species, pollen tube growth was evaluated at three positions: 1- pollen grains germinated on stigma, 2- pollen tubes visible at half the length of the style, 3- pollen tubes in the ovary reaching ovules (Figure 2). The number of pollen grains/pollen tubes was quantified for each position according to the criteria: 0- no germinated pollen grains/tubes 1- up to 10 germinated pollen grains/tubes, 2- several germinated pollen grains/tubes 3- dozens to hundreds germinated pollen grains/tubes.

### Seed morphology, germination and plant production

Seeds were collected at maturity 30–60 days after pollination depending on the seed parent. The obtained seeds were evaluated regarding their morphology under the stereomicroscope (Leitz DMRD, Leica, Germany). To determine germination percentage, 60 seeds were placed in transparent plastic germination boxes (12×8×5 cm, L×W×H) on moist filter paper (grade: 3 W, Munktell Filter AB, Grycksbo, Sweden). The remaining harvested seeds were sown in peat (Pindstrup Substrate no. 1, Pindstrup Mosebrug A/S). Seedling survival was determined one month after sowing. Plants were then transplanted and grown in 11 cm pots with peat in the greenhouse (22/18 ± 4°C day/night). Plants were kept under long day conditions (16/8 h, day/night). Mean number of seeds per capsule, germination percentage, number of seedlings obtained, seedling survival and number of plants and hybrids were recorded.

### Statistical analysis of phenotypic traits

The morphological evaluation of parental plants and pollen quality: the significance of differences was determined using one-way analysis of variance followed by Tukey’s honestly significant difference test (HSD) in the SPSS 22.0 for Windows statistical software package (SPSS Inc., Chicago, IL, USA).

For the analysis of pollen tube growth *in situ*, each of the five wild species used as maternal plants, the three paternal species (the two *K. blossfeldiana* cultivars and self-pollination of maternal plants) were compared in a one-way analysis of variance (ANOVA) for each time point (24 h, 48 h) followed by a pairwise comparison by a t-test. The same analysis was performed for reciprocal crosses, i.e. when five wild species and the two *K. blossfeldiana* cultivars were used as paternal plants, thus comparing the different maternal plants. All these analyses used R software version 3.0.3.

Simple and multiple linear regressions were used to relate the viability, germinability and genetic distance to the numbers of pollen tubes obtained after interspecific pollinations using R software version 3.0.3. Also this analysis was carried out for the two time points separately, with the 29 means, one from each cross, used as response variables.

## Results

### Morphological evaluation of parent plants

Pistil and style length were measured in the period of stigma receptivity, since the styles of the species from the Bryophyllum section elongate during flower maturation. Both carpels and style lengths were significantly different (*P* ≤ 0.05) between the species with much longer carpels and styles in the species of the Bryophyllum section (Figure 1B). No significant differences were observed between the two cultivars of *K. blossfeldiana*. The lengths of the pistil and style were the highest for *K. pubescens* i.e. 31.6 mm ± 0.4 mm (mean and S.E.) and 24.0 mm ± 0.3 mm, respectively. The shortest pistil was observed for *K. blossfeldiana* ‘Jackie’ i.e. 8.3 mm ± 0.1 mm, whereas the shortest style was observed for *K. blossfeldiana* ‘0089A’ i.e. 2.4 mm ± 0.1 mm.

### Cytological analysis

Chromosomes were counted for all species used in the hybridization. Both cultivars of *K. blossfeldiana* were found to be tetraploid with 2n = 68. The other species exhibited chromosome numbers typical for the *Kalanchoë* genus of 2n = 34 (*K. pubescens*, *K. marnieriana* and *K. gracilipes*) and 2n = 68 (*K. nyikae* and *K. campanulata*). During examination also single cells with higher ploidy levels were observed that can be explained by the polysomic nature of *Kalanchoë* species [12].

### Genetic diversity of the parental *Kalanchoë* genotypes

To evaluate the genetic relations among genotypes and species used in interspecific crosses, the seven genotypes were analyzed using 10 AFLP primer combinations. The number of scored marker fragments per primer pair is shown in Additional file 1. In total 856 marker fragments were produced for the *Kalanchoë* species and *Cotyledon tomentosa* as outgroup. The genetic distance between the two *K. blossfeldiana* cultivars was 0.312. The minimal genetic distance between two species was 0.695 for both *K. blossfeldiana* cultivars and *K. nyikae*, whereas the maximal genetic distance observed for *K. blossfeldiana* ‘Jackie’ and *K. marnieriana* was 0.801 (Table 2).

**Figure 3:**
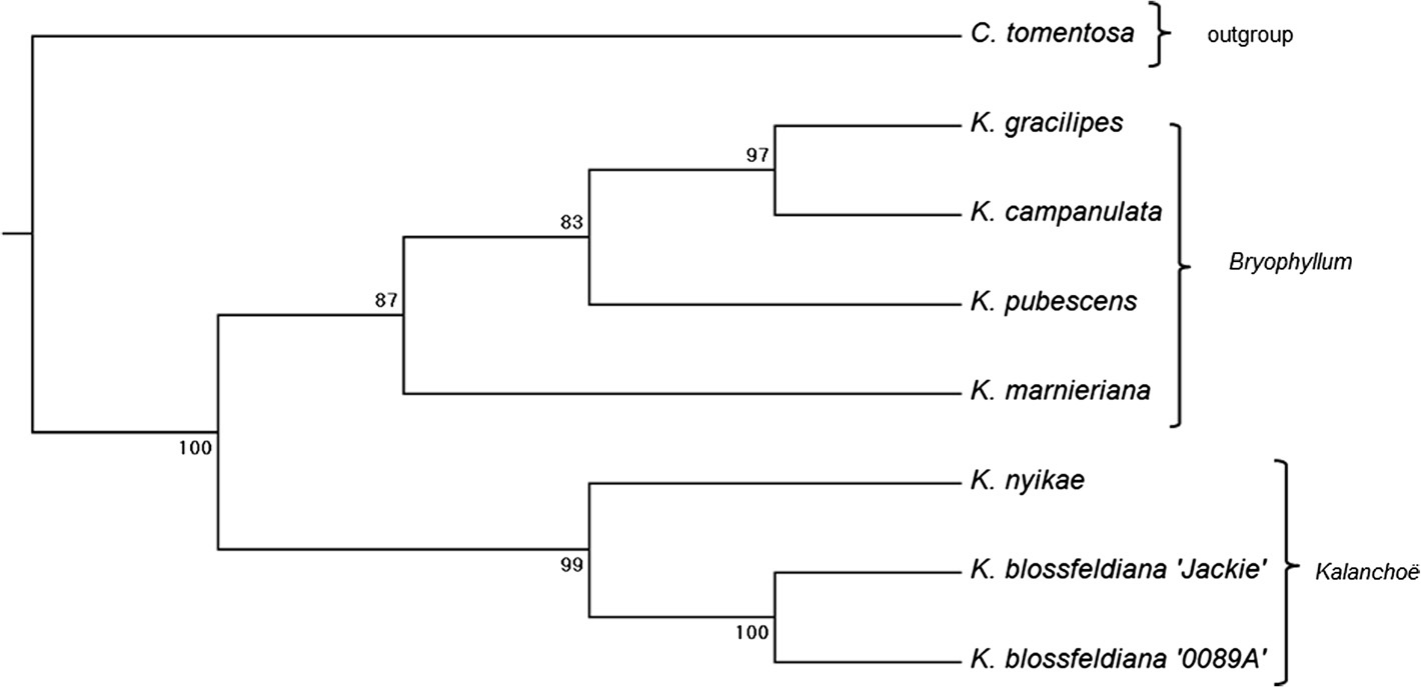
**Genetic relatedness of parental plants.** Neighbor-joining phenogram based on Jaccard similarity indexes computed from 856 AFLP markers for 7 *Kalanchoë* species and genotypes and *Cotyledon tomentosa* as the outgroup. The two sections of *Kalanchoë* genus: Kalanchoë and Bryophyllum are noted. The bootstrap percentages are shown next to the branches.

Based on the Jaccard similarity indexes a phenogram was calculated using the Neighbor-joining method. The phenogram differentiated two major clades representing the two sections of the *Kalanchoë* genus, Kalanchoë and Bryophyllum (Figure 3). The two species, *K. marnieriana* and *K. gracilipes,* previously classified to the separate Kitchinga section [22], grouped into a subcluster within the Bryophyllum section.

### Pollen quality

Pollen analyses were carried out to investigate if pollen quality was a limiting factor in the hybridization process. The level of pollen viability and germination ability differed significantly among the species. The highest level of pollen viability was observed for *K. blossfeldiana* ‘0089A’ i.e. 92% ± 3% (mean and S.E.), whereas the lowest level of pollen viability was detected for *K. blossfeldiana* ‘Jackie’ 35% ± 9%. The highest level of pollen germinability was observed for *K. pubescens* i.e. 67% ± 5%. *K. gracilipes* exhibited the lowest level of pollen germinability i.e. 19% ± 2%. The results of the two methods differed significantly for three genotypes: *K. blossfeldiana* ‘0089A’, *K. nyikae* and *K. gracilipes*. Thus, the data demonstrate that not all pollen grains, which were viable according to the staining test, were able to germinate (Figure 1C).

### Pollen tube growth

Pollen tube growth was examined in carpels following the intra- and interspecific crosses sampled 24 h and 48 h after pollination. Already after 24 h pollen tubes were detected in the ovary of all *Kalanchoë* species, thus the theoretical time of fertilization was determined to be less than 24 h.

The analysis of the numbers of pollen tubes presented similar patterns for 24 h and 48 h. Only in self-pollinations of *K. pubescens* a significant increase in the number of pollen tubes was detected in the ovaries 48 h after pollination compared with 24 h (Additional file 2, Figure 4).

**Figure 4:**
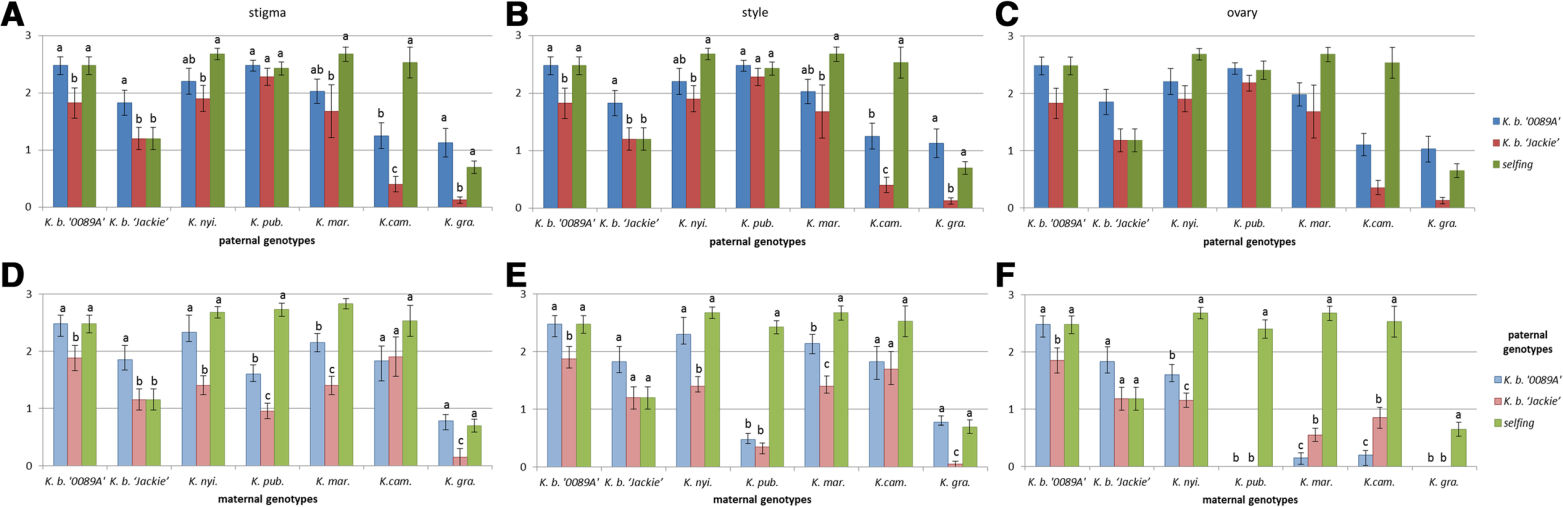
**Comparison of pollen tube numbers following interspecific crosses of** ***K. blossfeldiana*** **cultivars and self-pollinations. (A, B, C)** Number of pollen tubes after pollinations of two *K. blossfeldiana* cultivars with pollen of different *Kalanchoë* species and self-pollinations of paternal plants as controls observed on stigma, in the style and ovary. Values presented are means (±S.E.), n = 10. Values followed by different letters are significantly different (*P* ≤0.05) within the same maternal genotype according to t-test. **(D, E, F)** Number of pollen tubes after pollinations of *Kalanchoë* species with two *K. blossfeldiana* cultivars and self-pollinations of maternal plants as controls observed on stigma, in the style and ovary. Values presented are means (±S.E.), n = 10. Values followed by different letters are significantly different (*P* ≤0.05) within the same paternal genotype according to t test. The analyses were performed 48 h after pollination.

In the carpels sampled 48 h after self-pollinations, high numbers of pollen tubes were detected in the ovaries (Figure 4, green bars). The only exceptions were observed for *K. blossfeldiana* ‘Jackie’ and *K. gracilipes*, where numbers of pollen tubes were lower.

When *K. blossfeldiana* cultivars were used as maternal plants, the levels of pollen tubes in stigmas, styles and ovaries exhibited the same pattern (Figure 4A,B and C). Whereas, the reciprocal crosses (Figure 4D,E and F) showed decreased numbers of pollen tubes in the style (cross with *K. pubescens:* compare Figure 4B and E *K. pub.*). Even more dramatic decline of pollen tube numbers was observed in the ovaries following all inter-sectional crosses (Figure 4F).

The numbers of pollen tubes observed in crosses with *K. blossfeldiana* generally showed higher values when using *K. blossfeldiana* ‘0089A’ as a pollen donor (Figure 4D-F, blue vs red bars). However, in the crosses where *K. marnieriana* and *K. campanulata* were used as maternal plants, pollinations with *K. blossfeldiana* ‘Jackie’ resulted in significantly higher numbers of pollen tubes in the ovaries (Figure 4F).

**Figure 5:**
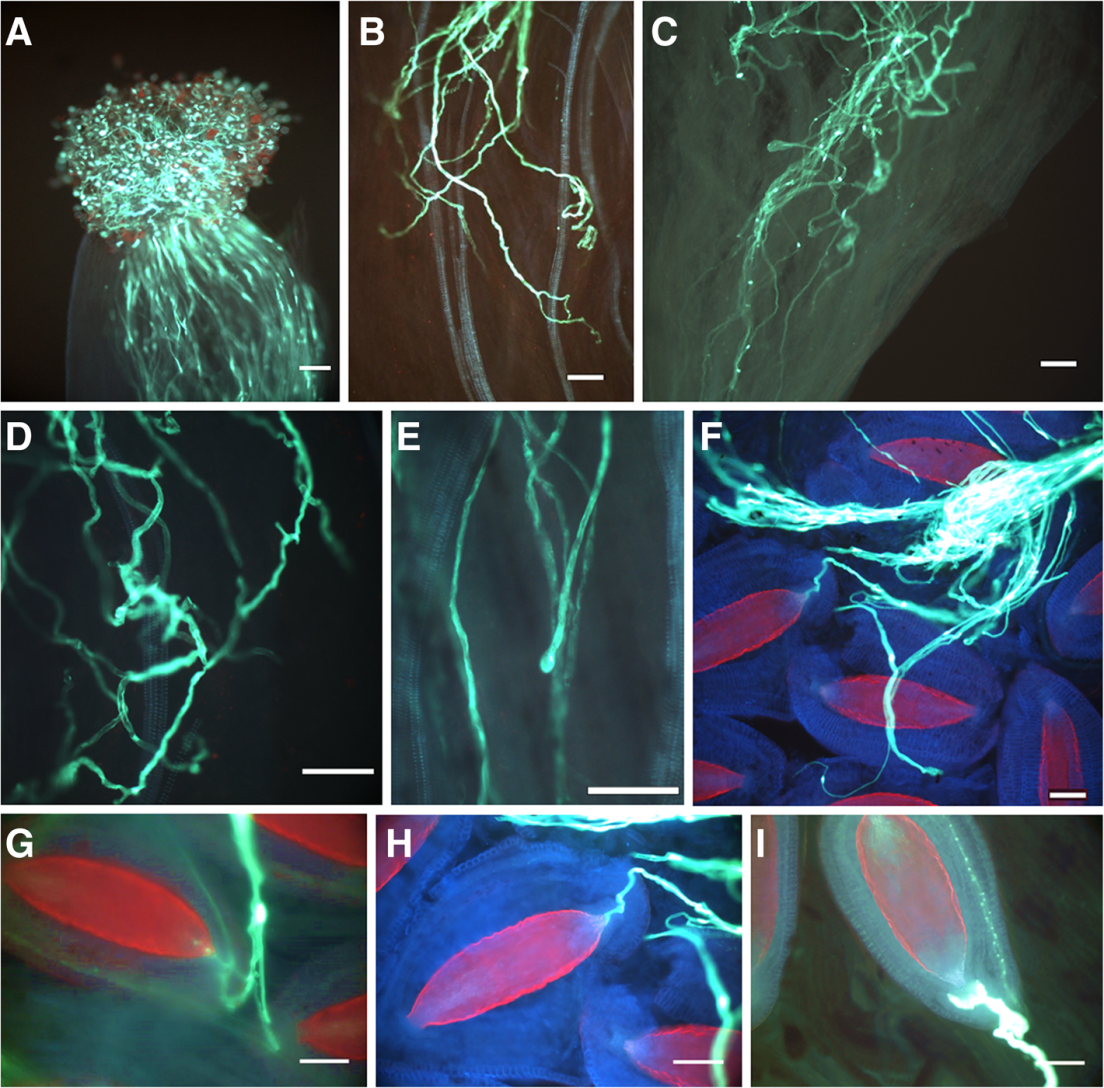
**Pollen tube growth and fertilization of** ***Kalanchoë*** **species. (A)** Pollen grains of *K. pubescens* on stigma of *K. blossfeldiana* ‘Jackie’ 24 h after pollination (a.p.). **(B)** Branching of pollen tube, *K. campanulata* x *K. blossfeldiana* ‘0089A’, 24 h a.p. **(C)** Stopped in growth pollen tubes of *K. blossfeldiana* ‘0089A’ in the style of *K. pubescens,* 24 h a.p. **(D)** Spiky pollen tubes, *K. campanulata* x *K. blossfeldiana* ‘0089A’, 24 h a.p. **(E)** Swelling tip of pollen; *K. gracilipes* x *K. blossfeldiana* ‘Jackie’, 48 h a.p. **(F)** Pollen tubes of *K. pubescens* in the ovary of *K. blossfeldiana* ‘0089A’, 48 h a.p. **(G)** Pollen tube of *K. campanulata* penetrating the ovule of *K. blossfeldiana* ‘0089A’, 48 h a.p. **(H)** Pollen tube of *K. pubescens* penetrating the ovule of *K. blossfeldiana* ‘Jackie’, 48 h a.p. **(I)** Pollen tube of *K. nyikae* penetrated the ovule of *K. blossfeldiana* ‘0089A’, 24 h a.p. *Scale bars*: 100 μm.

Qualitative analysis following intraspecific crosses revealed no abnormalities in pollen tube growth for the majority of the examined flowers (data not shown). An abnormal development was only detected in self-pollinations of *K. blossfeldiana* ‘Jackie’ and in the cross between *K. blossfeldiana* ‘Jackie’ and *K. blossfeldiana* ‘0089A’. Analysis of pollen tube growth following interspecific hybridization revealed different types of abnormalities that occurred in all examined cross-combinations. They included branching of the pollen tube (Figure 5B), spiky pollen tubes (Figure 5D), swelling of the tip (Figure 5E) and disorientation of pollen tubes.

Microscopic analysis also revealed that penetration of ovules by the pollen tubes occurred for all cross-combinations when *K. blossfeldiana* ‘0089A’ was used as maternal plant (Figure 5F-I). For *K. blossfeldiana* ‘Jackie’ ovule penetration was observed in crosses with *K. nyikae*, *K. pubescens* and *K. marnieriana*, and pollen tubes next to the ovules in other cross-combinations. When *K. blossfeldiana* plants were used as pollen donor, penetration of ovules was observed only in crosses with *K. nyikae*. In general, the observation of ovule penetration by pollen tubes suggests that fertilization takes place.

### Correlation analyses

The regression analysis of pollen tubes observed after 48 h showed significant correlation between numbers of pollen tubes germinated on the stigma with all three tested factors i.e. viability (*P* ≤ 0.01), germinability (*P* ≤ 0.001) and genetic distance (*P* ≤ 0.01) in simple regression analyses and with germinability (*P* ≤ 0.01) and genetic distance (*P* ≤ 0.05) in multiple regression analysis. The strongest correlation was demonstrated for germinability (Table 3). The number of pollen tubes observed in the style was also significantly related to all three factors (*P* ≤ 0.05, *P* ≤ 0.01 and *P* ≤ 0.05, respectively) in simple regression analysis and to germinability (*P* ≤ 0.05) and genetic distance (*P* ≤ 0.05) in multiple analyses. The correlation observed for the number of pollen tubes in the ovary was weaker, showing significant correlation to germinability and genetic distance (both *P* ≤ 0.05) in simple and multiple regression analysis. The number of pollen tubes observed in the style and the ovary was strongly related to the numbers of pollen tubes germinating on the stigma (Table 3). Furthermore, when the numbers of pollen tubes observed on stigma were added as explanatory variable in the multiple linear regression the three other explanatory variables were on the border of significance.

**Table 3:** **Relation between number of pollen tubes and pollen viability, germinability and genetic distance**

	**Stigma**				**Style**				**Ovary**			
	**Simple [%]**		**Multiple [%]**		**Simple [%]**		**Multiple [%]**		**Simple [%]**		**Multiple [%]**	
Viability	23.4^1^	**^2^		NS^2^	18.1	*^2^		NS	4.27	NS		NS
+stigma^3^					(91.2)	(***)						
Germinability	36.5	***	51.0	**	28.3	**	40.5	*	14.3	*	31.9	*
+stigma					(91.3)^4^	(***)^4^			(66.6)	(***)		
Genetic distance	22.4	**		*	18.4	*		*	13.8	*		*
+stigma					(91.1)	(***)			(64.5)	(***)		

^1^r^2^ value; ^2^NS, ***, **, *indicate non-significant, significant at *P* ≤ 0.001, *P* ≤ 0.01 and *P* ≤ 0.05, respectively; ^3^stigma as an explanatory variable; ^4^r^2^ and significance value for linear model with stigma as an explanatory variable.

The analysis of number of pollen tubes was performed 48 h after pollination.

### Seed morphology, seed set, germination and hybrid plant production

**Figure 6:**
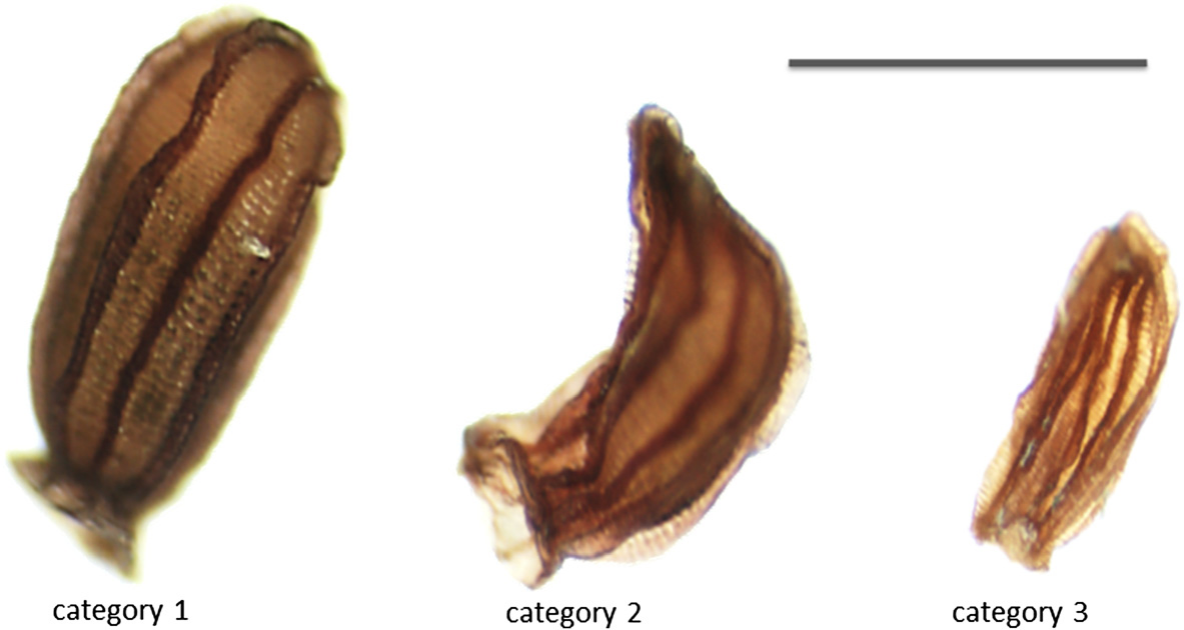
**Three types of seed morphology observed after intra- and interspecific crosses in** ***Kalanchoë.*** Category 1: normal seeds containing fully developed endosperm and embryo, germination; category 2: wrinkled seeds containing not fully developed endosperm, no germination; category 3: seed-like structure with no sign of endosperm and embryo, no germination. *Scale bar*: 1 mm.

The morphology of the obtained seeds was evaluated after harvesting. We distinguished three categories of seeds that could be found in *Kalanchoë* capsules (Figure 6). Category 1 included normal looking seeds that in general were able to produce plants. This category was found in all control crosses, except self-pollination of *K. gracilipes*. For interspecific crosses, category 1 was obtained in crosses of *K. blossfeldiana* cultivars with *K. nyikae,* both directions, and in crosses between *K. blossfeldiana* cultivars with *K. pubescens* and *K. marnieriana,* when wild species were used as pollen donors (Table 2). Category 2 included smaller and wrinkled seeds. This category was found for all crosses where *K. blossfeldiana* cultivars were used as maternal plants, as well as in the crosses where *K. nyikae* was used as maternal plant. Category 3 was seed-like structures with no sign of endosperm and embryo. It was found in all cross-combinations, including inter- and intraspecific crosses. Both category 2 and 3 seeds did not germinate (data not shown).

After harvesting, seed set per capsule was analyzed. The number of obtained normal seeds (category 1 see Figure 6) was generally higher for intraspecific than interspecific crosses (Table 2). In intraspecific hybridization self-pollination of *K. gracilipes* yielded no normal seeds and self-pollination of *K. blossfeldiana* ‘Jackie’ yielded in average only 5 seeds per flower. Seed set from interspecific crosses was generally low with the exception for the cross *K. blossfeldiana* ‘0089A’ x *K. nyikae* (28.2 ± 4.7 seeds per capsule; mean and S.E.)*.* For other interspecific crosses, the number of seeds ranged from 0.2 ± 0.2 and 0.1 respectively for *K. nyikae* x *K. blossfeldiana* ‘Jackie’ and *K. blossfeldiana* ‘Jackie’ x *K. marnieriana* to 6.0 ± 1.7 for *K. blossfeldiana* ‘Jackie’ x *K. nyikae*.

Germination percentages did not reveal any specificity for intra- or interspecific hybridization. They ranged from 37% for the cross between *K. blossfeldiana* ‘0089A’ and *K. marnieriana* to 98% for self-pollinations of *K. pubescens* and *K. campanulata* (Table 2).

Seedling survival was generally higher for intraspecific crosses ranging from 83 to 100% than for interspecific crosses ranging between 49 and 78%, with the exception of the cross between *K. blossfeldiana* ‘0089A’ and *K. marnieriana* where seedling survival was 95% (Table 2).

### Hybrid identification

**Figure 7:**
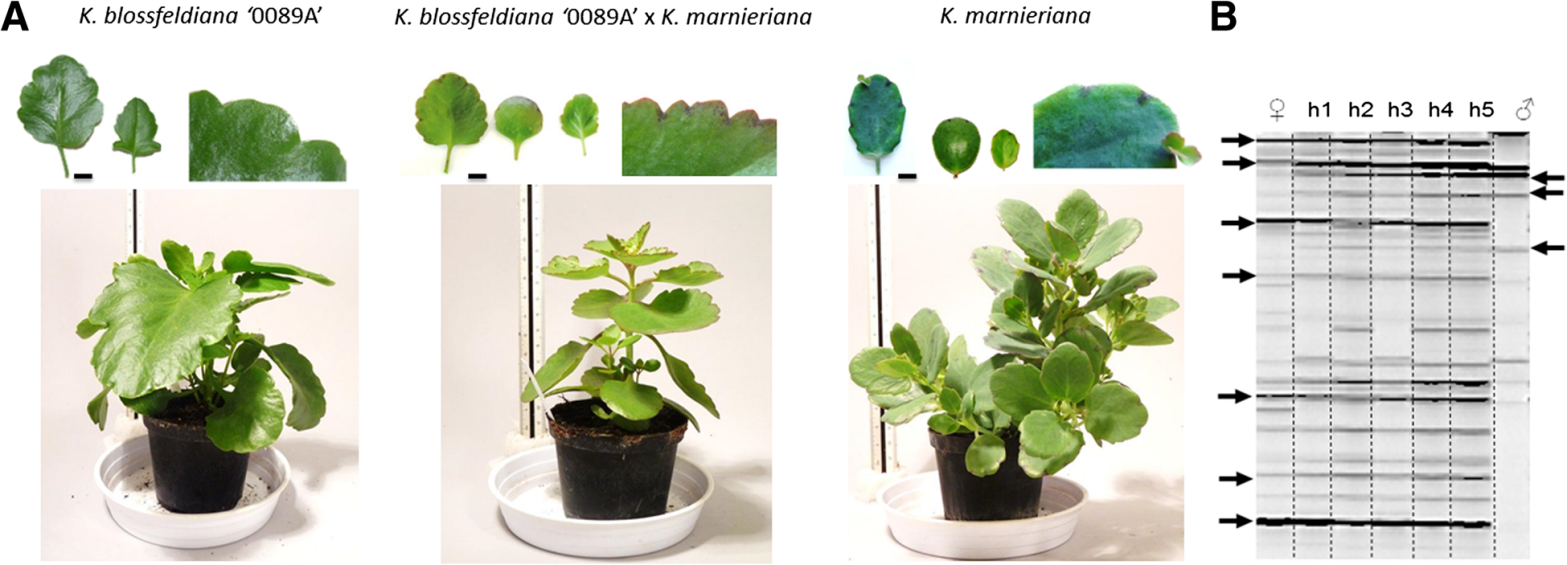
**Morphology and molecular analysis of interspecific hybrids. (A)** Leaf and plant morphology of maternal parent (left), hybrid (middle) and paternal parent (right) *Scale bars*: leaves: 2 cm. **(B)** AFLP banding pattern of 5 interspecific hybrids (h1-h5), amplified with primer combination *Hind*III -AAC/*Mse*I –TAG; some of the characteristic parental fragments are indicated with arrows.

Morphological hybrid identification was based on the examination of the progeny compared to parental plants. The hybrids obtained in this study had intermediate phenotype between the parents regarding plant architecture and leaf morphology (Figure 7A) as well as flower morphology (data not shown). Hybrids between smooth-leaf cultivars of *K. blossfeldiana* and hairy plants of *K. pubescens* exhibited formation of short hairs on the surface of the leaves. Plants obtained from the crosses among *K. blossfeldiana* ‘0089A’ and *K. marnieriana* inherited the purple spots on the leaf margin. The interspecific hybrids of *K. blossfeldiana* with *K. marnieriana* and *K. pubescens* showed more vigorous growth than both parents. Moreover, some genotypes of hybrids among *K. blossfeldiana* ‘0089A’ and *K. pubescens* exhibited formation of violet spots on the leaf margin and surface that were not observed in any of the parental plants (data not shown).

Based on the morphology of the progeny crosses, *K. blossfeldiana* ‘0089A’ x *K. nyikae, K. blossfeldiana* ‘Jackie’ x *K. nyikae, K. blossfeldiana* ‘0089A’ x *K. pubescens* and *K. blossfeldiana* ‘Jackie’ x *K. pubescens* resulted in 100% hybrid progeny. For the cross combination *K. blossfeldiana* ‘0089A’ x *K. marnieriana* and *K. nyikae* x *K. blossfeldiana* ‘0089A’ 25.8% and 9.2% of the obtained seedlings, respectively, were identified to exhibit hybrid characteristics (Table 2). The rest of the progeny displayed mother-like phenotypes.

AFLP analysis was carried out on 5 randomly selected individuals per cross-combination, which exhibited intermediate characteristics, to confirm hybridity on the molecular level. Band pattern was evaluated and all analyzed plants were verified as hybrids, including the intraspecific cross between *K. blossfeldiana* cultivars. One example of confirmation of hybrid status is presented in Figure 7B.

## Discussion

Many years of breeding with a limited number of species and narrow focus on targeted traits has resulted in restricted gene pool in the current *Kalanchoë blossfeldiana-*derived cultivars [9]. The utilization of wild species has a great potential for genetic improvement of existing breeding material. There are, however, different pre- and post-fertilization barriers hindering hybrid development. In our study extensive crosses among different *Kalanchoë* species and cultivars were performed in order to elucidate the nature of hybridization barriers, to relate them to the genetic distances and to develop novel interspecific hybrids.

### Pre-fertilization barriers

Pollen-pistil interactions are complex series of cellular and molecular interactions occurring between the diploid gynoecium and the haploid male gametophyte [23]. The pistil tissue provides guidance and nutrients that support pollen tube germination and growth; at the same time the pistil ensures a protection of ovules from access of unsuitable pollen [24]. It was suggested that plants with wet stigma surface, as the one occurring in *Kalanchoë* [25], have indiscriminate adhesion that relies only on liquid surface tension [24]. However, germination and pollen tube growth can be influenced by interactions between pollen and pistil.

In the present investigation we observed abundant growth of pollen tubes into ovary following self-pollinations of *Kalanchoë* species. This indicates a lack of the self-incompatibility system in the *Kalanchoë* genus. Self-pollinations of *K. blossfeldiana* ‘Jackie’ as well as *K. gracilipes* exhibited a significantly lower numbers of pollen tubes compared to other *Kalanchoë* species (Figure 4A, green bars). The low viability of the pistilate parent can be a reason for the reduced pollen tubes numbers, as demonstrated in distant crosses of *Tulipa* species [26].

In our study, examination of pollen germination and growth following interspecific pollination revealed a number of abnormalities. There are different factors in pollen-pistil interactions that influence formation and growth of pollen tubes such as composition of stigmatic exudates [27], water gradient potential in stigma [24], concentration gradients of Ca^2+^ ions [27], concentration of γ-aminobutyric acid, lipid molecules and proteins in the style [24]. Thus, species-specific differences regarding these factors can influence the mutual recognition and affect pollen germination and growth.

The quality of pollen used in artificial pollinations is one of the factors strongly affecting the success of pollination. Here, we used two methods to assess pollen quality: acetocarmine staining and *in vitro* germination test (Figure 1C). The results of germinability were significantly correlated to the numbers of pollen tubes on the stigmas, while viability was less or not significantly correlated (Table 3). These observations agree with the general opinion that *in vitro* pollen germination analysis is much more accurate for assessment of pollen quality than stain-based methods.

The flowers of *Kalanchoë* species are characterized by pronounced differences in the length of pistils and styles, especially among species belonging to different sections (Figure 1B). When *K. blossfeldiana* cultivars were used as paternal plants, the numbers of pollen tubes decreased in styles and ovaries when compared to stigma (Figure 4D-F). This finding agrees with previous results of interspecific hybridization of *Kalanchoë,* where in intersectional crosses, the hybrids were only obtained, when *K. blossfeldiana* was used as a maternal plants [12]. An analogous unilateral incongruity took place in distant crosses between different *Rhododendron* species [28]. However, other factors underlying this unilateral incongruity cannot be excluded.

The qualitative analysis of pollen tubes demonstrated atypical growth of pollen tube in wide crosses (Figure 5) suggesting improper interactions between pistil tissues and pollen tubes. Similar aberrations occurred in interspecific crosses in e.g. *Rhododendron* [29], *Dendranthema* [30], *Abelmoschus* [31] and *Vigna* [32] or the Bromeliaceae family [33]. Thus, this can be an additional evidence for disturbed pollen-pistil interactions occurring among *Kalanchoë* species.

### Post-fertilization barriers

It is often expected that crosses between individuals possessing the same chromosome numbers are more successful. In our study, interspecific hybrids were obtained following both intra- and interploidy crosses. Consistently, unsuccessful crosses did not exhibit specificity to ploidy level of parental plants. There are other genera in which species show a high level of cross-compatibility between different basic ploidy levels or chromosome numbers. In *Salvia* [34] and *Brassica* [35] hybrids from several cross-combinations including plants with different chromosome numbers were obtained. Interploidy hybrids were also obtained e.g. in *Chrysanthemum* [2] and *Dianthus* [36].

In our studies we observed abnormal wrinkled seeds that occurred with higher frequencies in interspecific crosses than in control pollinations (data not shown) indicating the occurrence of post-fertilization barriers connected to endosperm development. This also agrees with a previous study of interspecific hybridization in the *Kalanchoë* genus, where hybrids were only obtained following embryo rescue in specific cross-combinations [12].

The abnormal development of endosperm following interspecific crosses is highly similar to that observed in interploidy crosses. The abnormalities of endosperm development can include under- or overproliferation and changes in timing of cellularization. In general, it was postulated that a proper ratio of maternal and paternal genetic material in the endosperm is required for its normal development [37]. A later research on genomic imprinting in plants has suggested that the abnormal development of endosperm may be caused by non-equivalency of imprinted genes. Although the epigenetic modification on parental genes should be complementary within a species, it is possible that the pattern of epigenetic modification and expression of imprinted genes are different in distant species [37].

The genetic differences between cultivars are a plausible explanation for diverse capacity for hybrid production. A similar situation was observed in *Petunia* [38] and *Dianthus* [3]. *K. blossfeldiana* ‘0089A’ was more suitable as hybridization partner than cultivar ‘Jackie’, both in terms of seed and plant production (Table 2).

### The influence of the genetic distance on plant cross-compatibility

In our study we used AFLPs to determine genetic relations among the *Kalanchoë* species. The neighbour-joining phenogram obtained from the analysis of 856 markers (Figure 3) revealed a similar clustering compared to previously obtained phenograms from RAPD and ITS data [39,40]. All analyses were able to separate the species in the different sections of *Kalanchoë.* Previous analyses distinguished three main intrageneric clusters that coincide with taxonomic division according to Boiteau [22] that divides the *Kalanchoë* genus into three sections. In our analysis, the species are separated into two main clusters that coincide with the Bryophyllum and Kalanchoë sections. The species classified into the former section of Kitchingia form a subcluster within Bryophyllum. This situation can be explained by the low number of species included in our analysis. Another reason for the slightly different architecture of the constructed phenogram may be a selection of an outgroup that can influence the position of species in the main clusters.

The genetic distances estimated in this study are relatively high (0.312 – 0.801) (Table 2). The previous analysis by Gehrig et al. [39] that used RAPD markers reported genetic distances in the same range as reported here. The probable reason for the high level of genetic diversity within the *Kalanchoë* genus may be a rapid evolutionary development of this taxon [8].

The general theory postulates that difficulties of obtaining interspecific hybrids increase with the phylogenetic distance between parental plants [41]. In the case of plant populations, however, this rule is not necessarily true [42]. In our study, the connection between genetic distances obtained from genotyping using AFLP markers and reproductive isolation exhibited only rough correlation. The obtained results revealed a significant correlation (*P* ≤ 0.05) between the numbers of pollen tubes on the stigma and parental divergence. This may indicate heterospecific differences in composition of stigmatic exudate, or in size and shape of pollen and stigma morphology (data not shown) as it was suggested in *Helleborus* species [43]. As a consequence, the production of seeds and subsequently plants from interspecific crosses of *Kalanchoë* was more successful within section than inter-sectional crosses (Table 2), similarly to results obtained for *Helleborus* [43].

### Hybrid characteristics and molecular verification

The progeny obtained after interspecific pollinations exhibited clear intermediate character for several traits (Figure 7A). The formation of viviparous plants, however, which is a typical trait of the Bryophyllum section*,* was not observed in the hybrids. This agrees with results of intersectional crosses among *K. spathulata* and *K. laxiflora,* [13,43] and *K. blossfeldiana* and *K. pubescens* [12] where formation of viviparous plants in hybrids was not detected. The findings suggest that this trait is recessive or quantitative [13].

Following the two cross-combinations i.e. *K. nyikae* x *K. blossfeldiana* ‘0089A’ and *K. blossfeldiana* ‘0089A’ × *K. marnieriana* only 9.2% and 25.8%, respectively, exhibited the intermediate characteristics between both parental plants. The rest of the progeny displayed mother-like phenotypes. A possible explanation for this situation can be an accidental contamination during the pollination. However, unpollinated flowers left as controls on the same maternal plants did not set any seeds. Another possible explanation for mother-like plants is the production of seeds via apomixis [6,44].

## Conclusion

The present work has elucidated details of hybridization barriers occurring during interspecific hybridization within the *Kalanchoë* genus. Several hybridization barriers have been identified to take place in both the pre-and the post-fertilization phase. The results suggest that both quality of pollen and style length are strong factors influencing the hybridization success. Furthermore, the cross-compatibility between *Kalanchoë* species is influenced by the genetic background of hybridization partners and genetic distance.

Future breeding efforts should take into consideration the occurrence of unilateral incongruity between *Kalanchoë* species. Moreover, the abnormal seed development following distant crosses, probably due to endosperm degeneration, suggests utilization of embryo rescue techniques in further attempts to develop new interspecific hybrids.

Two new interspecific hybrids between *K. nyikae* as maternal plants and *K. blossfeldiana* as well as *K. blossfeldiana* and *K. marnieriana* were produced for the first time in our study. Thorough morphological analysis will be performed to evaluate the commercial value of these novel hybrids.

## Additional files

Additional file 1:
**Scored AFLP marker fragments and levels of polymorphism.**
Additional file 2:
**Comparison of pollen tube numbers following interspecific crosses of** ***K. blossfeldiana*** **cultivars and self-pollinations.** (A, B, C). Number of pollen tubes after pollinations of two *K. blossfeldiana* cultivars with pollen of different *Kalanchoë* species and self-pollinations of paternal plants as controls observed on stigma, in the style and ovary. Values presented are means (±S.E.), n = 10. Values followed by different letters are significantly different (P ≤0.05) within the same maternal genotype according to t-test. (D, E, F) Number of pollen tubes after pollinations of *Kalanchoë* species with two *K. blossfeldiana* cultivars and self-pollinations of maternal plants as controls observed on stigma, in the style and ovary. Values presented are means (±S.E.), n = 10. Values followed by different letters are significantly different (P ≤0.05) within the same paternal genotype according to t-test. The analyses were performed 24 h after pollination.
