# Supplementary material for: Evaluation of reproductive barriers contributes to the development of novel interspecific hybrids in the Kalanchoë genus
Source: BMC Plant Biol. 2015 Jan 21;15:15. doi: 10.1186/s12870-014-0394-0 (PMC4352236; doi:10.1186/s12870-014-0394-0)
Supplement: Additional file 1: — Scored AFLP marker fragments and levels of polymorphism. [file 12870_2014_394_MOESM1_ESM.pdf]

Additional file 1: Table S1. Scored AFLP marker fragments and levels of polymorphism

*Hind*III/ *Mse*I primer pairs	Total no. of scored fragments		Polymorphic fragments within the genus *Kalanchoë* [%]
-AAC/-ACC	85		81.2
-AAC/-ATT	111		91.9
-AAC/-CCA	90		97.8
-AAC/-CCC	86		93.0
-AAC/-TAA	83		92.8
-AAC/-TAG	119		95.0
-AAC/-TAT	50		94.0
-AAT/-AAC	92		90.2
-AAT/-CCA	58		89.7
-AAT/-CCC	82		96.3
Σ	856	mean	92.3
