# Supplementary material for: Evaluation of reproductive barriers contributes to the development of novel interspecific hybrids in the Kalanchoë genus
Source: BMC Plant Biol. 2015 Jan 21;15:15. doi: 10.1186/s12870-014-0394-0 (PMC4352236; doi:10.1186/s12870-014-0394-0)
Supplement: Additional file 2: — Comparison of pollen tube numbers following interspecific crosses of K. blossfeldiana cultivars and self-pollinations. (A, B, C). Number of pollen tubes after pollinations of two K. blossfeldiana cultivars with pollen of different Kalanchoë species and self-pollinations of paternal plants as controls observed on stigma, in the style and ovary. Values presented are means (±S.E.), n = 10. Values followed by different letters are significantly different (P ≤0.05) within the same maternal genotype according to t-test. (D, E, F) Number of pollen tubes after pollinations of Kalanchoë species with two K. blossfeldiana cultivars and self-pollinations of maternal plants as controls observed on stigma, in the style and ovary. Values presented are means (±S.E.), n = 10. Values followed by different letters are significantly different (P ≤0.05) within the same paternal genotype according to t-test. The analyses were performed 24 h after pollination. [file 12870_2014_394_MOESM2_ESM.pdf]

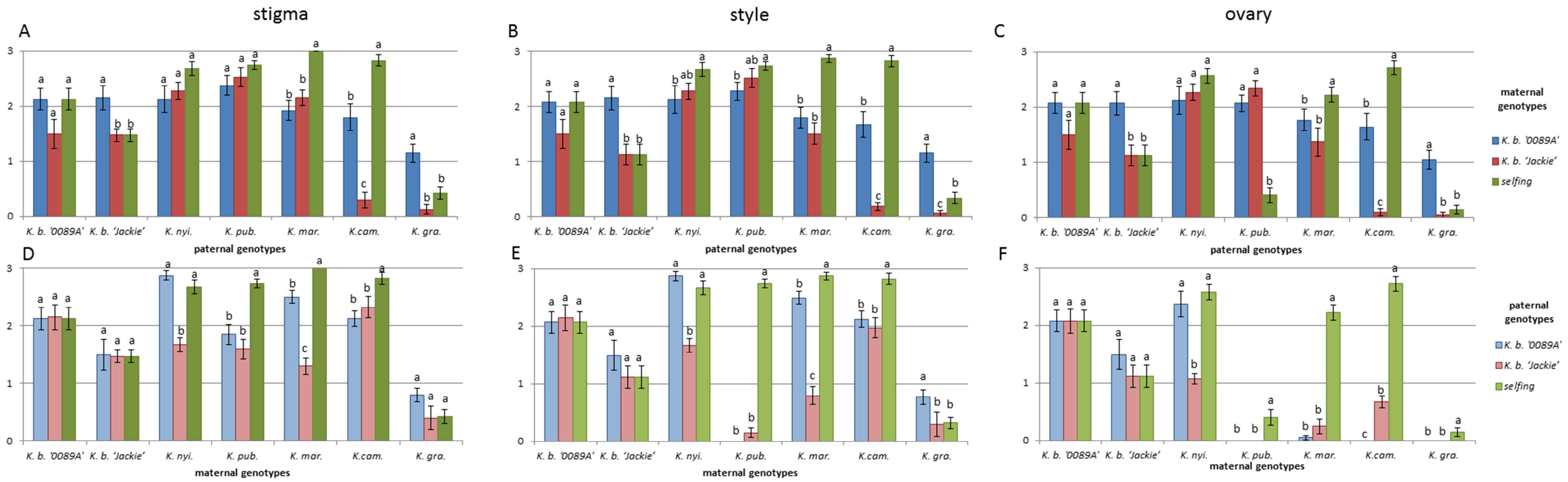

Comparison of pollen tube numbers following interspecific crosses of *K. blossfeldiana* cultivars and self-pollinations. (A, B, C) Number of pollen tubes after pollinations of two *K. blossfeldiana* cultivars with pollen of different *Kalanchoë* species and self-pollinations of paternal plants as controls observed on stigma, in the style and ovary. Values presented are means (±S.E.), n=10. Values followed by different letters are significantly different (P <0.05) within the same maternal genotype according to t-test. **(D, E, F)** Number of pollen tubes after pollinations of *Kalanchoë* species with two *K. blossfeldiana* cultivars and self-pollinations of maternal plants as controls observed on stigma, in the style and ovary. Values presented are means (±S.E.), n=10. Values followed by different letters are significantly different (P <0.05) within the same paternal genotype according to t test. The analyses were performed 24 h after pollination.
